# Nuclear Factor-Kappa B Activity Regulates Brain Expression of P-Glycoprotein in the Kainic Acid-Induced Seizure Rats

**DOI:** 10.1155/2011/670613

**Published:** 2011-02-21

**Authors:** Nian Yu, Qing Di, Hao Liu, Yong Hu, Ying Jiang, Yu-kui Yan, Yan-fang Zhang, Ying-dong Zhang

**Affiliations:** ^1^Department of Neurology, Nanjing Brain Hospital, Nanjing Medical University, 264 Guangzhou Road, Nanjing, Jiangsu 210009, China; ^2^Department of Neurology, University of Pittsburgh School of Medicine, Pittsburgh, PA 15213, USA; ^3^Department of Neurology, Huzhou Central Hospital, Huzhou, Zhejiang 313000, China

## Abstract

This study was aimed to investigate the effect of NF-**κ**B activity on the seizure susceptibility, brain damage, and P-gp expression in kainic acid- (KA-) induced seizure rats. Male SD rats were divided into saline control group (NS group), KA induced epilepsy group (EP group), and epilepsy group intervened with NF-**κ**B inhibitor-pyrrolidine dithiocarbamate salt (PDTC group) or with dexamethasone (DEX group). No seizures were observed in the rats of NS group. Compared with NS group, increased P-gp expression and NF-**κ**B activation in the rat brain of the EP group were observed after KA micro-injection. Both PDTC and DEX pre-treatment significantly increased the latency to grade III or V seizure onset compared to EP group but failed to show neuron-protective effect as the number of survival neurons didn't significantly differ from that in EP group. Furthermore, PDTC pre-treatment significantly decreased P-gp expression along with NF-**κ**B activation in the hippocampus CA3 area and amygdala complex of rats compared with the EP group, implying that NF-**κ**B activation involved in the seizure susceptibility and seizure induced brain P-gp over-expression. Additionally, DEX pre-treatment only decreased P-gp expression level without inhibition of NF-**κ**B activation, suggesting NF-**κ**B independent pathway may also participate in regulating seizure induced P-gp over-expression.

## 1. Introduction

Even though much progress has been made on the therapeutic intervention for epilepsy, approximately 1/3 of epilepsy patients who have been treated with 2 or more antiepileptic drugs (AEDs) at the maximal tolerate dose are still suffering from refractory epilepsy (RE) [1, 2]. Multidrug resistance (MDR), which is one of the major causes of clinical drug-therapy failure for epilepsy, was reported to be mediated by P-glycoprotein (P-gp) overexpression, which has been determined in human epileptogenic brain tissue resected during epilepsy surgery and in brain of rats following the prolonged status epileptic insults [3]. P-gp functions as active pumps at the endothelial cell membrane of blood-brain barrier (BBB) to prevent AEDs from reaching their targets in the brain [4]. Therefore, inhibition of brain P-gp overexpression under the epileptic conditions may be a potential approach for RE treatments in the future. However, the precise mechanisms underlining this brain P-gp overexpression remain unclear.

P-gp is a protein encoded by the gene *multidrug resistance1 *(*MDR1*) in human and *mdr1a* and *mdr1b* in rodents, and it is expressed widely in epithelial cells of many organs including the brain, liver, intestine, kidney, adrenal gland, and testes [5]. It has been reported that the expression level and the activity of P-gp on the BBB was related to central nervous system (CNS) or peripheral inflammation [6]. Since epilepsy is also considered as a chronic inflammatory condition, one would expect there might be a link between inflammation and brain P-gp overexpression under epilepsy conditions. In support of this hypothesis, Bauer et al. reported that P-gp expression level was increased by extracellular glutamate at concentrations similar to the epileptic brain, and this effect was mediated through the N-methyl-D-aspartic acid (NMDA) receptor and cyclooxygenase-2 (COX-2) activation [7–9], as this effect was blocked either by the COX-2 inhibitors, such as celecoxib, SC-58236, and NS-398 or in the COX-2 knock-out mice. 

Nuclear factor-kappa B (NF-*κ*B), a key regulator of immune and inflammatory response, has been implicated in the neuropathological processes of seizure and epilepsy [10, 11]. The most widely characterized NF-*κ*B subunits in CNS are p50 and p65 [12]. Once activated, NF-*κ*B translocates to the nucleus and binds to the promoter region of target genes by recognizing the DNA *κ*B consensus elements. The target gene products of NF-*κ*B include many cytokines and chemokines, cell adhesion molecules, and immunoreceptors [13]. Previous studies have shown that inflammation can regulate the expression of P-gp through NF-*κ*B signaling pathway at the BBB under several pathological conditions, such as infection, tumor, and diabetes mellitus (DM) [14–16]. Thus we hypothesized that the same signaling pathway may also be responsible for the upregulation of P-gp expression in the epileptic brain.

In the current paper, we investigated the expression level of P-gp using a rat seizure model provoked by intrahippocampal microinjection of kainic acid (KA). Two distinct NF-*κ*B inhibitors, pyrrolidine dithiocarbamate salt (PDTC), and dexamethasone (DEX) were administrated to the seizure rats to block NF-*κ*B activation. Besides the P-gp expression, the rat mortality, seizure susceptibility, and brain damage were also analyzed. Our results demonstrated that NF-*κ*B inhibition attenuated the overexpression of P-gp in the KA-induced seizure rats and decreased seizure susceptibility without affecting the rats' mortality and brain damage. 

## 2. Materials and Methods

### 2.1. Animals

Adult male Sprague-Dawley rats weighing 140 ± 20 g were purchased from Slack Shanghai Laboratory Animal co., Ltd, (License No: (Shanghai) 2007-0005). Before treatment in the experiments, all the rats were housed for 2 weeks at constant temperature 22 ± 1°C and relative humidity (60%) and had free access to standard food and water under a fixed 12 h light/dark cycle. Procedures involving animals and their care were conducted in conformity with the Guidance Suggestions for the Care and Use of Laboratory Animal, formulated by the Ministry of Science and Technology of China [17]. 

### 2.2. Experimental Reagents and Instrument

Kainic acid was purchased from Sigma (Louis, Mo, USA). Pyrrolidine dithiocarbamate (PDTC), a specific NF-*κ*B activity inhibitor, was purchased from International Laboratory (USA). Dexamethasone Sodium Phosphate (DEX) was from Jinyao (Tianjin, China). Monoclonal rabbit anti-Glucose transporter 1 (GLUT-1) and antineuronal nuclear antigen (NeuN, MAB377) antibody were from Chemicon (USA). Monoclonal anti-P-glycoprotein (C219) antibody was purchased from Abcam (England). Polyclonal anti-NF-*κ*B p65 (Catalog No.: 10745-1-AP) antibody was purchased from ProteinTech Group, Inc (USA) and antiphospho-NF-*κ*B p65 antibody (Ser276, Cat No.: BS4135) was from Bioworld Technology, Inc. (USA). TUNEL-based In Situ Apoptosis Assay Kit (Cat: KGA7025) was from KeyGEN (Nanjing, China). Lycra medical image acquisition and analysis system was purchased from Leica (Germany). Stereotactic apparatus (H12070515) was from Huaibei Zhenghua (Anhui, China). 

### 2.3. Seizure Induction with KA

Fifty rats were randomly divided into four groups, (1) saline control group (NS group, *n* = 8), (2) KA induced epilepsy group (EP group, *n* = 14), (3) epilepsy intervened with PDTC pretreatment (PDTC group, *n* = 14), and (4) epilepsy intervened with DEX pretreatment (DEX group, *n* = 14). After being anesthetized with 10% chloralhydrate (3.5 mL/kg) by intraperitoneally injection (i.p.), each rat was placed on the stereotaxic frame. A hole was drilled in the skull with dental reamers. Using a microinjector, seizure were induced by stereotactic-injection of 3 *μ*L KA (0.5 mg/mL) into the CA3 region of the left hippocampus area (5.0 mm posterior to the bregma, 5.0 mm left lateral from the midline, and 5.0 mm deep from the dura) according to the atlas of Paxions and Wastson [18]. Rats in the PDTC group and DEX group were pretreated with PDTC (150 mg/kg) or DEX (0.4 mg/kg), respectively by i.p injection for NF-*κ*B inhibition 30 min prior to KA microinjection; while the NS group and EP group rats received equivalent normal saline injection as a control [19]. After surgery, the scalps were sutured. Seizure activity was assessed using behavioral observation and seizure scoring according to Racine Scale [20]: 0, no reaction; I, stereotype mounting, eye blinking, and/or mild facial clonus; II, head nodding and/or several facial clonus; III, myoclonic jerks in the forelimbs; IV, clonic convulsions in the forelimb with rearing; and V, generalized, clonic convulsions associated with loss of balance. Only rats that reached grade IV seizure were selected for further analysis. Seizures were terminated after 90 min by administration of 10% chloralhydrate and repeated as needed.

### 2.4. Observation of Seizures

Behavior study was performed by 3 independent observers who were blinded to the sample identity. According to the literature [21], the time ranged from the initial injection of KA to grade III and V seizure onset (the seizure onset time, denoted SOT3 and SOT5) was recorded, respectively, as the latency index of the model rats to evaluate seizure susceptibility. 

### 2.5. Tissue Processing

Twenty-four hours after seizure, models were established successfully, rats were perfused transcardially with saline followed by 4% paraformaldehyde. The brains were rapidly removed and fixed in 10% formalin phosphate buffer for 24 h. The brain tissues were fixed in 10% formalin neutral buffer for 24 h, followed by dehydration with alcohol, clearing infiltration, and paraffin embedding within 4 h*．*

### 2.6. Immunohistochemistry

After being fixed in acetone, two or three sections of the following brain regions were chosen for immunohistochemistry study: −2.3 mm to −3.8 mm relative to bregma, including cerebral cortex, piriform cortex, hippocampus CA3/CA4, dentate gyrus, amygdale complex, and substantia nigra pars reticulata. CA3/CA4 was defned as the CA area found in the hilus of the dentate gyrus. Briefly, slides were incubated with 0.03% H_2_O_2_ in PBS for 10 min at room temperature (RT), followed by 15 min incubation with 5% normal goat serum in PBS. Sections were then incubated with different primary antibodies: anti-NF-*κ*B p65 (1 : 40), anti-p-NF*κ*B-p65 (S276) (1 : 200), anti-P-gp (1 : 50), and anti-NeuN (1 : 400) in 3% bovine albumin serum in PBS at 4°C overnight. Immunoreactivity was tested with the avidin-biotin-peroxidase technique, using 3,3′-diaminobenzidine (DAB) as the chromogen. Sections were dried, and then dehydrated in graded alcohols, and mounted on the slides. 

For the immunofluorescence study, the transverse sections were blocked and then incubated in an antibody mixture with antibodies against P-gp (C219, 1 : 100) and GLUT-1 (1 : 1000). The slides were then washed for three times followed by incubation with TRITC or FITC conjugated secondary antibodies. Fluorescent signals from double-labeled sections were detected with a confocal microscope (Leica TCS SP2; Leica, Benstein, Germany) and the images were processed with Adobe Photoshop 6.0. 

### 2.7. TUNEL Assay

TUNEL staning was performed as previously described with some modifications [22]. Briefly, slides were firstly incubated with 0.03% H_2_O_2_ in PBS for 10 min at room temperature, followed by proteinase K treatment (7.5 *μ*L of 20 *μ*g/*μ*L Proteinase K in 150 mL 10 mM Tris, pH 8.0). After rinsed twice with 1XPBS, the slides were then incubated with the TUNEL reaction mixture (or control label solution for negative control) to label the DNA fragments in the cell with the digoxigenin-nuclotide. FITC-conjugated antidigoxigenim antibody was then applied to detect the TUNEL-positive cells with fluorescence microscopy. 

### 2.8. Cell Counting

Cell counting was performed by 3 independent observers who were blinded to the sample identity. For each rat, the positive cells were counted in 5 fields of each slide with a 40x objective lens. The representative high-power nonoverlapping fields of the interesting areas were captured using an Olympus Fluor view laser scanning confocal microscope. The areas examined included the hippocampus CA3 area, dentate gyrus, and amygdaloid nuclear complex. Data obtained in each field per slice were added together to make a single final data for each slice. Values of two slices per rat were averaged and this value was used to calculate the percentage of NeuN, NF-*κ*B, or P-gp positive cells. A frequency score was assigned for each cell-type using 4 distinct grades: I, ≤25% (rare); II, 26%–50% (sparse); III, 51%–75% (high); IV, >75% (all). Although this cell counting method has some limitations as compared to design-based stereological analysis [23], the occurrence of any bias in counting neurons or other cells should similarly affect control and experimental samples since these samples underwent the same methodological procedures in parallel. 

### 2.9. Data Analysis

Where applicable, data were presented as means ± standard deviation, and the expression levels of NF-*κ*B and P-gp were described as ranked data. Statistical analysis was carried out using the SPSS software (13.0). Statistical differences were evaluated by ANOVA followed by the LSD posttest for multiple comparisons. Ranked data were evaluated by Kruskal-Wallis test and Wilcoxon rank sum test. A *P* value below  .05 was considered statistically signifcant. 

## 3. Results

### 3.1. The Seizure Susceptibility of kainate-Treated Rats after NF-*κ*B Inhibition

**Figure 1:**
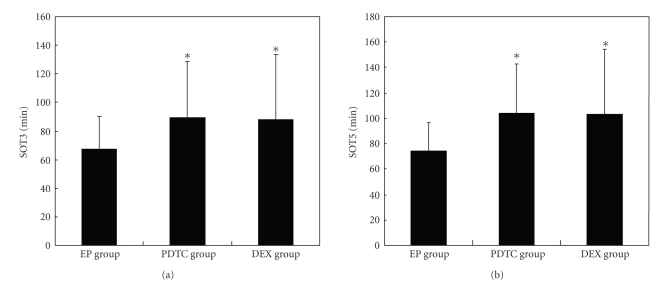
PDTC and DEX pretreatment prolonged the seizure onset time in KA induced rats. Mean values for the seizure onset time to grade III (SOT3, A) and grade V (SOT5, B) as the latencies to seizure of each group. SOT3 in the PDTC and DEX pretreated groups was 89.58 ± 39.28 min and 87.92 ± 45.80 min, respectively, compared to 67.50 ± 22.91 min in EP group. SOT5 in the PDTC and DEX pretreated groups was 104.17 ± 39.07 min and 103.33 ± 51.27 min, respectively, compared to 74.58 ± 22.10 min in EP group. (*P < .05, versus EP group).

No seizures were observed in the rats of NS group. About 86% of KA micro-injected rats (36 out of 42) developed status epilepticus (SE), which matched to the study criterion. Each of EP, PDTC, and DEX group has 2 rats died of severe seizures after KA injection. Finally there were 12 rats in the EP group, PDTC group, and DEX group respectively, and 8 rats in the NS group. As shown in Figure 1, the seizure latencies of rats pretreated with PDTC or DEX were prolonged in contrast with EP group. The mean value for the seizure onset time to grade III (SOT3) was 89.58 ± 39.28 min and 87.92 ± 45.80 min for the PDTC and DEX groups, respectively, compared to 67.50 ± 22.91 min for the EP group. Similarly, SOT5 in the PDTC and DEX pretreated groups was 104.17 ± 39.07 min and 103.33 ± 51.27 min, respectively, significantly longer than 74.58 ± 22.10 min for the EP group. 

### 3.2. Cell Lose in kainate-Treated Rats after NF-*κ*B Inhibition

**Figure 2:**
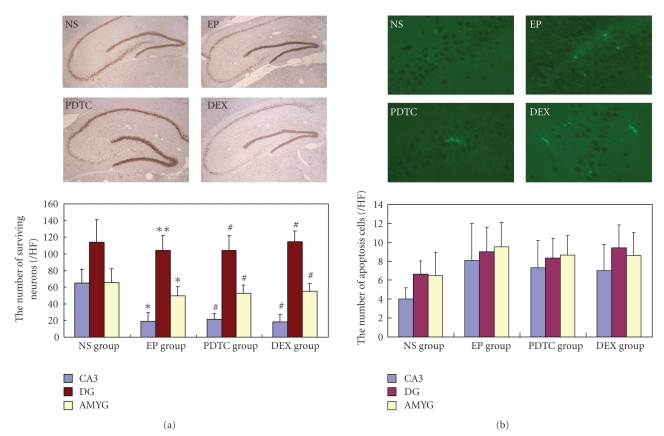
PDTC and DEX pretreatment failed to prevent KA induced brain cell loss. (a) Representative photos of immunohistochemistry staining with anti-NeuN antibody (×100). Immunohistochemistry staining was performed to check the surviving neurons in the brain. The shape and number of neurons is normal in NS group (NS), while significant neuronal loss in the CA3 area was observed 24 h after KA micro-injection in the EP group (EP). In the KA induced rat pre-treated with PDTC (PDTC) or DEX (DEX), the survival neuron numbers did not significantly increase compared to the EP group. Additionally, the neuron number in dentate gyrus was not significantly changed among the four groups. Histogram showed the number of surviving neurons in the indicated brain area of each group (/HF). *P < .05 versus NS group; **P < .01 versus NS group; ^#^ *P* > .05 versus EP group, CA3, hippocampus CA3 area; DG: dentate gyrus; AMYG: amygdaloid nuclear. (b) Representative photos of TUNEL staining (times 200). Cells undergoing apoptosis were detected (labeled by FITC and shown in green) in each group 24 h after KA or vehicle injection. KA injection did not increase TUNEL positive cells significantly; there was no difference in TUNEL positive cell numbers between PDTC/DEX and EP groups. CA3, *P* = .052; DG, *P* = .65; AMYG, *P* = .061, by ONEWAY-ANOVA test; CA3, hippocampus CA3 area; DG: dentate gyrus; AMYG: amygdaloid nuclear.

In the control NS group, the pyramidal cells of hippocampus CA3 area revealed a typical 4 to 5 layers structure with closely aligned cells and clear nuclei. However, in the EP group, 24 h after KA injection, the brain structures were markedly damaged, evident by tissue edema as well as neuronal degeneration and loss in the CA3 area of hippocampus and amygdaloid nuclear complex. The number of surviving neurons was 64.75 ± 16.89/HF, 114.00 ± 26.87/HF, and 65.75 ± 16.34/HF in the CA3 area, dentate gyrus and amygdaloid nuclear complex in NS group rats, respectively. Compared to the NS group, the numbers of survival neurons in the hippocampus CA3 area (19.00 ± 10.18/HF, *P* = .000) and amygdaloid nuclear complex (49.42 ± 11.20/HF, *P* = .017) significantly declined in the EP group (*P* < .05), except for the dentate gyrus area (104.50 ± 17.38/HF, *P* > .05). Although PDTC (21.25 ± 6.98/HF, 104.42 ± 17.46/HF, 52.83 ± 9.77/HF) and DEX (18.50 ± 8.65/HF, 114.67 ± 12.63/HF, 55.26 ± 9.41/HF) pretreatment shows a slightly neuron-protective effect compared to the EP group, it did not reach statistical significance (*P* > .05). Furthermore, no significant structure changes could be observed in the dentate gyrus among the three KA induced groups (Figure 2(a)). 

In order to detect cells undergoing apoptosis in the brain tissue, TUNEL assay was performed. As shown in the Figure 2(b), a few TUNEL-positive cells were scattered in the brains of NS group rats. 24 h after KA micro-injection, the TUNEL-positive cell numbers did not increase significantly compared to the NS group (*P* > .05). In addition, no significant difference in TUNEL staining was observed between DEX group and KA group, or between PDTC group and KA group (*P* > .05). These results indicate that the KA induced brain cell loss is primarily through a necrotic mechanism instead of apoptosis mechanism (Figure 2(b)). 

### 3.3. The Brain Expression of NF-*κ*B p65 and P-gp

**Figure 3:**
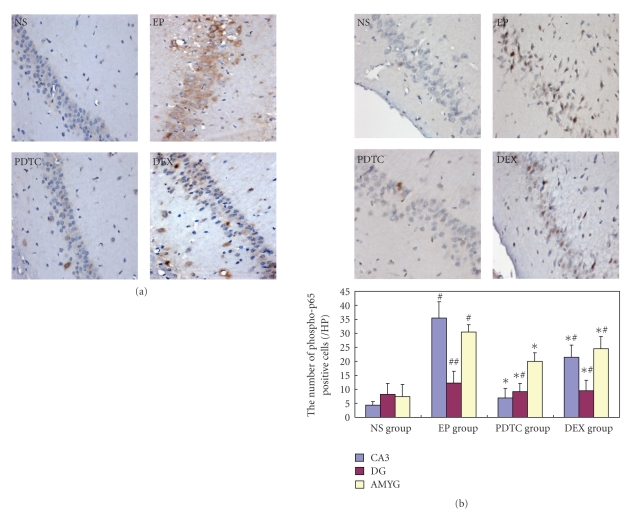
Pretreatment of PDTC, but not DEX, decreases KA induced NF-*κ*B activation. (a) Representative photos of immunohistochemistry staining with anti- NF-*κ*B p65 antibody (×400). NF-*κ*B expression and distribution pattern was detected in the rat brains of each group 24 h after KA or vehicle micro-injection. The NF-*κ*B p65 immunoreactive cell number increased as well as more nuclear staining was observed in the EP group (EP) compared to the control rat (NS). By contrast with EP group, the number of NF-*κ*B p65 positive cells were decreased in the group pre-treated with PDTC (PDTC), but not in the group with DEX pretreatment (DEX). (b) Representative photos of immunohistochemistry staining with antiphospho-NF-*κ*B p65 antibody (×400). Activated NF-*κ*B in the rat brains of each group was detected 24 h after KA or vehicle micro-injection. The number of Phospho-p65 positive cells significantly increased 24 h after KA injection (EP) compared with that in NS group. Compared to the EP group, activated NF-*κ*B signal was significantly decreased in hippocampus CA3 area and amygdaloid nuclear complex of the rats pre-treated with PDTC (PDTC), but not in the rats with DEX pretreatment (DEX). ^#^ *P* < .05 versus NS group; ^##^ *P* > .05 versus NS group; *P < .05 versus EP group; ^∗#^ *P* > .05 versus EP group CA3, hippocampus CA3 area; DG: dentate gyrus; AMYG: amygdaloid nuclear.

**Figure 4:**
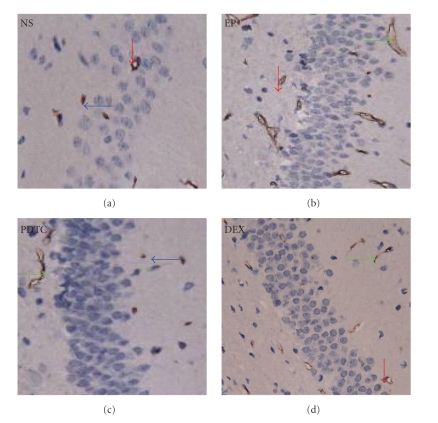
Pretreatment of PDTC and DEX both decreases KA induced P-gp overexpression. P-gp expression was detected by immunohistochemistry staining (×400) with an anti-P-gp antibody. A few P-gp positive cells were observed in the control NS group (NS). The number of the P-gp immunoreactive cells significantly increased in the KA micro-injected brain of EP group (EP), and it was mainly in vascular endothelial cells. By contrast with EP group, P-pg immunoreactive cells were decreased similarly in KA micro-injected brains pre-treated with PDTC (PDTC) or DEX (DEX). **↓: **Neurons; ***←*: **glial cells; **→: **vascular endothelial cells.

In NS group rats, only several NF-*κ*B p65 and P-gp immunopositive cells in the brain were detected. Compared to the NS group, the numbers of the NF-*κ*B p65 and P-gp positive cells significantly increased in the EP group. As shown in Figure 3(a), NF-*κ* B p65 was widely expressed in neurons, glial cells, and vascular endothelial cells in the EP group 24 h after KA micro-injection. In addition, the p65 signal was also frequently detected in the cell nuclei and more prominently in hippocampus CA3 area, suggesting the activation of NF-*κ* B in the epileptic brain. Meanwhile, the expression level of P-gp was also significantly increased in the EP group and the major positive cells were vascular endothelial cells in the brain. Compared to the EP group, the expression of NF-*κ*B p65 and P-gp was decreased in the PDTC group, and the location of NF-*κ*B p65 was changed to the cytoplasm instead of nuclei with shallow staining, suggesting a decreased NF-*κ*B activation by the PDTC pretreatment. Interestingly, in the DEX group, even P-gp expression was declined compared to the EP group; the expression level and distribution of NF-*κ*B p65 was not significantly different from the EP group as that happened in the PDTC pretreated epileptic rats. (Tables 1 and 2 and Figures 3(a) and 4). Collectively, the above data shows the level of NF-*κ* B activation is closely related with the expression level of P-gp in the KA induced epileptic brain. 

To confirm these findings, another antibody against activated NF-*κ* B, antiphospho- NF-*κ* B p65 (S276) was used in the immunohistochemistry. As shown in Figure 3(b), only a few phospho-p65 positive cells could be detected in the normal brain tissue; while the phospho-p65 positive cell number significantly increased in the EP group, confirming the activation of NF-*κ* B after KA micro-injection in brain. Similar to the above NF-*κ*B p65 staining result, phospho-p65 positive cell numbers also significantly decreased in the PDTC group compared to the EP group (CA3 and AMYG, all *P* = .000), and DEX pretreatment decreased phospho-p65 positive cell numbers compared to the EP group as well but did not reach statistic significance (*P* = .09, Figure 3(b)). 

**Figure 5:**
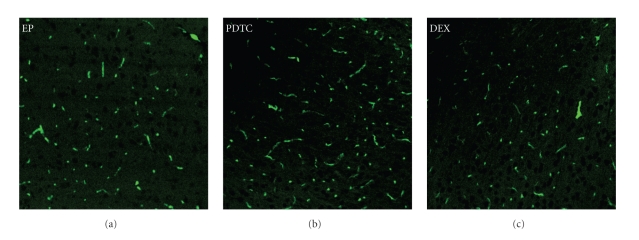
Pretreatment of PDTC and DEX did not change endothelial cell numbers compared with EP group. Representative photos of immuno-staining with anti-GLUT1 antibody (×200). Endothelial cells in the rat brains of each group were labeled with an anti-GLUT1 antibody paired with a FITC conjugated secondary antibody (shown in green). No significant difference in endothelial cell numbers could be detected between PDTC/DEX and EP group.

To exclude the possibility that the decreased P-gp staining in the PDTC and DEX group compared with EP group was due to cell loss, an immuno-fluorescence staining experiment was performed with an anti-GLUT1 antibody paired with FITC conjugated secondary antibody, which will specifically label endothelial cells in the tissue. As shown in Figure 5, endothelial cell number did not change significantly between PDTC and EP group or between DEX and EP group. As the previous NeuN staining also showed similar neuron numbers in these three groups, we think the decreased staining of P-gp in the rat brain of PDTC and DEX group was most likely due to the decreased expression level rather than the decreased cell numbers. 

## 4. Discussion

The *multidrug resistance (MDR)* gene encodes a family of proteins including P-gp that belong to the ATP-binding cassette (ABC) transporter family [24]. P-gp, which has been studied extensively, plays a critical role in the etiology of RE by limiting AEDs into their targets in the brain. Several traditional AEDs such as carbamazepine, Phenobarbital, and phenytoin are the transport substrates of P-gp [25]. P-gp brain expression is increased under prolonged seizure conditions such as status epilepticus or frequent spontaneous seizures [26], and selective inhibition of P-gp could enhance the brain uptake of AEDs and therefore improve their anticonvulsant effects [4, 5]. For example, P-gp specific inhibitors, such as tarquidar (TQD, XR9576) and PS833, were reported to improve the anticonvulsive effects of AEDs by competitively inhibiting P-gp transport activity [27]. However, these competitive inhibitors reverse MDR at concentrations that usually result in unacceptable toxicity in the clinical trials [28]. Recently, novel technologies, such as antisense oligonucleotides, ribozymes, and the RNA interference, have also been applied to block the upregulation of brain P-gp after epileptic assaults, but the clinical application of these techniques are still not practical [29]. Therefore, searching for other approaches to inhibit the seizure upregulated P-gp expression and functions are still necessary for the efficient RE patients' treatment.

Inflammation is an important biological process that is activated after SE or prolonged seizure [30]. Mounting evidence supports that inflammation not only contributes to epileptogenesis but also is involved in RE induced neuronal injury [31]. NF-*κ*B, a major regulator of inflammation, has been linked with epilepsy in many reports. Overexpression of NF-*κ*B in the brain hippocampus has been observed both in the experimental models of epilepsy [32] and in hippocampus tissues surgically removed from patients with temporal lobe epilepsy [33]. Yu et al. reported that NF-*κ*B mediated TNF-induced upregulation of *mdr1* promoter activity by selectively binding p65 to *mdr1*, suggesting inflammation enhances BBB efflux transport through NF-*κ*B activation [34]. However, whether P-gp overexpression in the epileptic brain is mediated through NF-*κ*B activation remains unclear. Therefore, the present study mainly focused on the relationship between NF-*κ*B activation and P-gp expression in the brain of KA induced seizure rats. To avoid the toxicity of systemic administration, we established a seizure rat model by using KA microinjection into the hippocampus area. Here we reported that, 24 h after the onset of KA induced seizures, the brain expression of P-gp was significantly increased in the hippocampus CA3 area and amygdala complex, which are important brain regions for epileptogenesis [35], and this effect was abolished in the rats pretreated with PDTC, a selective inhibitor of NF-*κ*B by blocking I*κ*B-ubiquitin ligase activity [36]. This data suggests that NF-*κ*B activation plays an important role in the upregulation of P-gp expression in the seizure brain. Our data is consistent with Bauer's research which showed that inhibition of NF-*κ*B by the SN50 peptide could block the inductive effect of the proinflammatory cytokines on P-gp upregulation in the isolated rat brain capillaries [37]. 

 Furthermore, our data with DEX treatment suggested some other NF-*κ*B independent pathway may also be involved in the upregulation of P-gp in the KA induced seizure rat brain, as DEX pretreatment decreased the P-gp expression without significantly inhibiting NF-*κ*B P65 expression in hippocampus CA3 and amygdala complex area. For example, some other transcription factors such as activator protein-1 (AP-1) [38] and pregnane xenobiotic receptor (PXR) have been reported to be also involved in the regulation of P-gp expression under many pathological conditions and their activation can be regulated by DEX [39]. Therefore, DEX may decrease the KA induced brain P-gp expression through regulation of these transcription factors instead of NF-*κ*B activation. Further experiments are needed to explore the precise mechanisms regarding the inhibition effect of DEX on the P-gp overexpression in the epileptic brain. On the other hand, it is noteworthy that the two NF-*κ*B inhibitors used in the current report, PDTC and DEX, also have many other pharmacology effects besides inhibition of NF-*κ*B signal pathway. For example, PDTC also has effect on nitric oxide synthetase and interfere with nitric oxide production. Therefore, further experiments with SiRNA technique to specifically block down NF-*κ*B p65 or p50 will be helpful to provide more direct and specific evidence about the role of NF-*κ*B activation in P-gp overexpression in the epileptic brain.

NF-*κ*B is a pluripotent nuclear transcripton factor implicated in the regulation of multiple cellular processes, including the inflammatory response, cell apoptosis and survival [40, 41]. However, the role of NF-*κ*B inhibition on seizure activity as well as seizure induced brain damage is still unclear and controversial. Shin et al. had reported that PDTC prevented hippocampal neuronal loss in the KA induced seizure model [42]; whereas Mattson's work showed that intrahippocampal injection of I*κ*B decoy DNA to block NF-*κ*B activation prior to KA administration enhanced neuronal cell death [43]. Recently, Lubin et al. [44] has reported that the seizure susceptibility of KA induced-seizure rats was increased after NF-*κ*B activity inhibition. The reasons for this discrepancy might be (1) different approaches were applied for the NF-*κ*B inhibition: recent study found that different regulator of NF-*κ*B could interfere with a specific subset of target genes' expression and then shows different effects on cell death or survival [43]; (2) distinct observation time points were chosen. In the present study, we found that the inhibition of NF-*κ*B by PDTC and DEX pretreatment failed to show neuron-protective effect in the KA induced epileptic brain as the number of survival neurons did not significantly differ from that in EP group. The possible reasons for the lack of neuron-protective effect of NF-*κ*B inhibition in our study might include (1) some NF-*κ*B independent mechanisms might mediate the KA induced neuron loss in our epilepsy model; (2) both PDTC and DEX also could affect other signal pathways besides the NF-*κ*B inhibition; (3) NF-*κ*B activation in this epilepsy model might even have some protection effect on the cell death. Further studies with NF-*κ*B SiRNA or I*κ*B overexpression would be more useful to check the role of NF-*κ*B activation in the epileptic brain's neuron loss. 

In the current study, even both PDTC and DEX pretreatment inhibit the brain overexpression of P-pg and decrease seizure susceptibility in our KA micro-injection induced seizure model, no significant changes regarding the seizure rats' mortality and brain damage were observed. This result suggests that the overexpression of P-gp in the seizure brain alone is not neuron-toxic; its deleterious effect in the developing of RE is mediated through the enhanced AED drug efflux. Further experiments to observe the index of seizure susceptibility, severity, and brain damage in KA induced seizure rats with a combined treatment of AED and PDTC or DEX would be of more clinical importance. 

## 5. Conclusions

Taken together, the present study reports that inhibition of NF-*κ*B activation by PDTC could block the seizure induced brain P-gp overexpression and extend the seizure onset time, implying NF-*κ*B activation involved in the seizure susceptibility and seizure induced brain P-gp overexpression. However, this inhibition did not change the seizure rats' brain damage and mortality. In addition, DEX reduced P-gp overexpression without significantly changing NF-*κ*B p65 expression, suggesting NF-*κ*B independent pathway may be also involved in the seizure induced P-gp overexpression. Further studies are needed to address whether inhibiting NF-*κ*B activation could enhance the efficacy of AEDs to overcome pharmacoresistance in a chronic drug-resistant rat model. 

##  Disclosure/Conflict of Interest

The authors declare no conflict of interest. 

## Figures and Tables

**Table 1:** NF-*κ*B p65 expression in each group (the number of cases).

groups	CA3 area				DG				AMYG			
	I	II	III	IV	I	II	III	IV	I	II	III	IV
NS group	7	1	0	0	8	0	0	0	5	3	0	0
EP group	1	4	2	5	2	4	4	2	1	3	2	6
PDTC group	8	4	0	0	7	5	0	0	3	6	3	0
DEX group	6	3	0	3	4	8	0	0	2	3	6	1
P*	*P* = .001				*P* = .000				*P* = .002			
EP versus NS	*P* = .001				*P* = .003				*P* = .001			
PDTC versus EP	*P* = .001				*P* = .006				*P* = .015			
DEX versus EP	*P* = .057				*P* = .025				*P* = .141			

Note: Values are presented as 4 distinct grades: I: ≤25% (rare); II: 26%–50% (sparse); III: 51%–75% (high); IV: >75% (all), according to the proportions of NF-*κ*B positive cells. *The overall difference among 4 groups using the Kruskal-Wallis test. DG: dentate gyrus; AMYG: amygdaloid nuclear.

**Table 2:** P-gp expression in the rat brain of each group (the number of cases).

groups	CA3 area				DG				AMYG			
	I	II	III	IV	I	II	III	IV	I	II	III	IV
NS group	7	1	0	0	8	0	0	0	8	0	0	0
EP group	0	2	9	1	2	4	6	0	2	3	6	1
PDTC group	7	5	0	0	10	2	0	0	7	5	0	0
DEX group	0	10	2	0	5	6	0	1	2	3	6	1
P*	*P* = .000				*P* = .000				*P* = .001			
EP versus NS	*P* = .000				*P* = .001				*P* = .001			
PDTC versus EP	*P* = .000				*P* = .001				*P* = .004			
DEX versus EP	*P* = .001				*P* = .058				*P* = .029			

Note: Values are presented as 4 distinct grades: I: ≤25% (rare); II: 26%–50% (sparse); III: 51%–75% (high); IV: >75% (all), according to the proportions of P-gp positive cells. *The overall difference among 4 groups using the Kruskal-Wallis test. DG: dentate gyrus; AMYG: amygdaloid nuclear.
